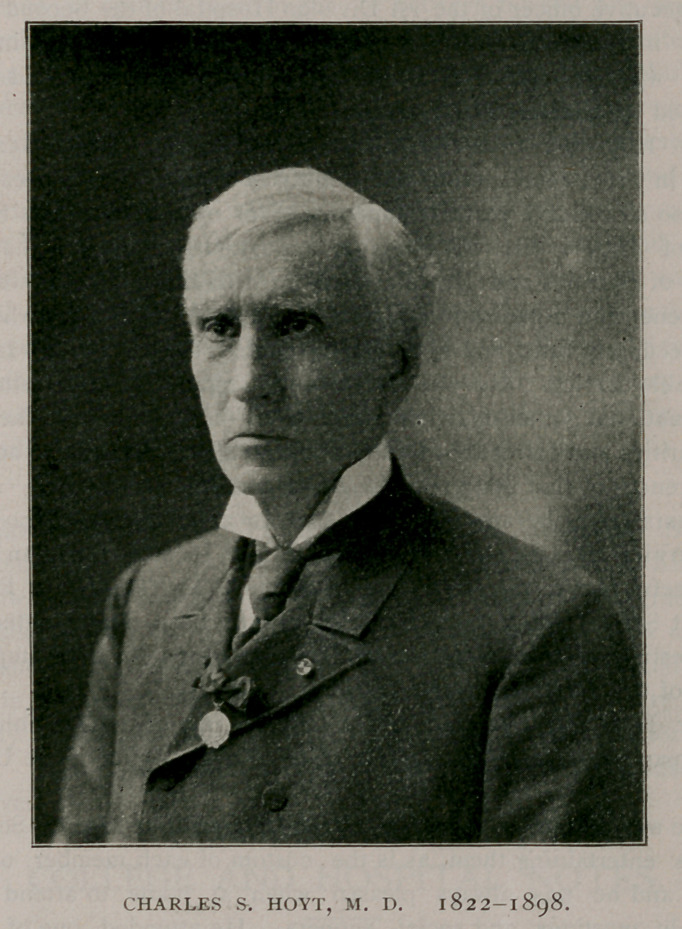# Dr. Charles S. Hoyt

**Published:** 1899-01

**Authors:** 


### OBITUARY.

Dr. Charles S. Hoyt, of Canandaigua, N. Y., died at his residence in that village, December 13, 1898, aged 76 years. The immediate cause of his death was pneumonia, though his general health of late had not been quite up to the robust standard that had

CHARLES S. HOYT, M. D. 1822–1898.

favored him all his life. He was born at Ridgefield, Conn., June 8, 1822, where he passed his childhood days, but in 1834 he came to Potter, Yates county, N. Y., and received his preliminary education at Rushville Academy. Upon attaining his majority he entered upon the study of medicine and took his doctorate degree at Geneva Medical College in 1847.

Dr. Hoyt entered at once upon the practice of his profession at Potter, where he was so engaged when at the outbreak of the Rebellion, in 1861, he was appointed by Governor Morgan, one of the war committee for the then 26th Senate district. He assisted in recruiting the 126th regiment, N. Y. Volunteers, and served as assistant surgeon of that organisation until May 11, 1864, when he was promoted to surgeon of the 39th N. Y. Regiment. He became the executive officer of the 1st Division Hospital of the Second Army Corps in June, 1864, and so continued until mustered out in July, 1865, when he returned to his home in Yates county, and there resumed the practice of his profession.

Dr. Hoyt was twice elected to the Assembly, first in 1852 and again in 1867, representing the County of Yates on both occasions. He also served five years as superintendent of public schools for the town of Potter. In 1868 he was appointed secretary of the state board of charities and henceforward devoted himself to the work of that beneficent organisation, finding in it a field of labor for which he was peculiarly fitted and in which he gained distinction for faithful and able service. He was prominently engaged in organising the great national gatherings, held annually, of those eminent in the work of charities and correction, and was repeatedly called upon to address conferences of that body on subjects of national importance. About two years ago he became superintendent, by appointment, of the board of charities, of state and alien poor, and had recently taken an active and useful part in the establishment of the Craig Colony for Epileptics, at Sonyea, N. Y. He had also of late been deeply interested in investigating the condition and devising means for their improvement of the New York State Indians.

Dr. Hoyt was a comrade of the Grand Army of the Republic and a companion of the military order of the Loyal Legion of the United States.

He was an interested member of the Canandaigua Medical Society, always entertaining them, as is the custom of each member, once a year, and he was always pleased when at home to attend their monthly meetings and social suppers. He attended one of these gatherings less than three weeks before his death. And so midst his busy official life he kept faith with the guild of medicine.

Dr. Hoyt was married in 1866 to Dora, daughter of Major Barnum, of Bristol, who survives, as do also three children: Charles S., Miss Agnes B., and Miss Jean B., all residents of Canandaigua. One brother, residing in Yates county, is also living.

Dr. Hoyt was preëminently a man of affairs. Very few men had a more extensive acquaintance in every county of the state, and he was also well known throughout the United States and in Europe. His official position brought him in contact with every condition of life, high and low, rich and poor, all of whom he served with the same degree of exact justice. It is doubtful if any person ever complained of ill-treatment at his hands, no matter how much difference of opinion there might be between himself and another. His amiable temperament, his sense of right, his sunny countenance and his sound judgment all conspired to make him a model official, an excellent citizen and a lovable man. His military service was most creditable. At the request of the writer he became executive officer of the field hospital of the first division of the Second Army Corps in the summer of 1864, which service has before been alluded to, but it is proper to add that for promptitude, for kind and skilful care of sick and wounded soldiers, Dr. Hoyt was without a superior in zeal and self-abnegation. From the Rapidan to Petersburg—May 3d–June 17, 1864—was a perpetual battle; fighting days and marching nights, was the great task as it was the great glory of the Army of the Potomac. He was never too late, always vigilant, faithful, enduring; his epitaph may fittingly so be written.

## Figures and Tables

**Figure f1:**